# Conservative treatment in adolescent idiopathic scoliosis with curves over 45°: is the measurement in Cobb degrees the only parameter to be considered?

**DOI:** 10.1186/1748-7161-9-S1-O23

**Published:** 2014-12-04

**Authors:** Angelo Gabriele Aulisa, Vincenzo Guzzanti, Marco Giordano, Francesco Falciglia, Marco Fuiano, Lorenzo Aulisa

**Affiliations:** 1 UOC Orthopaedic and Traumatology, Childrens Hospital Bambino Gesu, Rome, Italy; 2 Department of Orthopaedics Universita Cattolica del Sacro Cuore, Rome, Italy

## Background

The recent literature showed positive results in bracing patients with idiopathic scoliosis above 45° that refused surgery. However, no one has investigated whether other parameters are able to affect the results.

## Aim

The aim of this study was to evaluate the effectiveness of the brace in idiopathic scoliosis with curves above 45° and to assess whether the magnitude of the curve in Cobb degrees is the only parameter for the indication to surgical or conservative treatment.

## Design

This is a prospective study based on ongoing database including 1,238 patients with idiopathic scoliosis .

## Methods

The study including idiopathic scoliosis with 45° or more, Risser 0-4, who had utterly deny any surgical intervention. Fulfill the inclusion criteria 160 patients. Of these, 104 patients has definite outcome, 28 abandoned treatment and 28 are currently under treatment. The minimum duration of follow-up was 24 months. X-rays was used to to obtain Cobb degrees and torsion of the apical vertebra (Perdriolle's method). Three outcomes were distinguished in agreement with SRS criteria: curve correction, curve stabilization and curve progression. We have divided the sample in subgroup according to Risser (0-2; 3-4), to rotation (<20; >25) and to type of Curve. The Kruskal Wallis and Spearman Rank Correlation tests have been used as statistical tests.

## Results

The results from our study showed that the 104 patients with a definite outcome Cobb mean value was 47 ± 5.37 SD at beginning and 34.18 ±8.45 SD at follow-up. Perdriolle was 20.2 ± 5.49 SD at beginning and 16.76 ± 7.04 at follow-up. Curve correction was accomplished in 81 patients (77.8%), whereas a curve stabilization was obtained in 15 patients (14.4%), 9 patients (8.6%) have a curve progression and 16 (15.4%) where recommended for surgery. The subgroups with rotation <20 showed a correction/stabilization in 98.1% and surgery referral in 1.8% while in subgroups with rotation >25 a correction/stabilization was achieved in 69.4% but surgery referral in 60.8%. The subgroups with risser 0-2 showed a correction/stabilization in 92.6% and surgery referral in 10.3% while in subgroups with risser 3-4 a correction/stabilization was achieved in 91.6% but surgery referral in 25%.

## Conclusion

Results allow to say that an adequate conservative treatment must be absolutely considered in the treatment of scoliotic curves in patients who refuse surgery, in particular if the rotation is lower than 20 and Risser is between 0-2, the results will be better.

